# The Effect of Camphor on Sex Hormones Levels in Rats

**Published:** 2014-05-25

**Authors:** Sima Shahabi, Seyed Gholam Ali Jorsaraei, Ali Akbar Moghadamnia, Effat Barghi, Ebrahim Zabihi, Masoumeh Golsorkhtabar Amiri, Ghorban Maliji, Alieh Sohan Faraji, Maryam Abdi Boora, Neda Ghazinejad, Hajar Shamsai

**Affiliations:** 1 Cellular and Molecular Biology Research Center (CMBRC), Babol University of Medical Sciences, Babol, Iran; 2 Fatemeh Zahra Fertility and Infertility Reproductive Health Research Center, Babol University of Medical Sciences, Babol, Iran; 3 Department of Physiology and Pharmacology, Babol University of Medical Sciences, Babol, Iran; 4 Department of Anatomy, Babol University of Medical Sciences, Babol, Iran

**Keywords:** Camphor, FSH, LH, Testosterone

## Abstract

In some traditional therapies, it has been claimed that camphor (a crystalline ketone obtained from *cinnamomum camphora*) would be a suppressor of sexual behaviors and sex hormones. This study evaluated the effects of camphor on sex hormones, like luteinizing hormone (LH), follicle-stimulating hormone (FSH) and testosterone. In this experimental study, 56 male rats were divided into 5 groups, including control (n=12), sham (n=11) and three treatment groups (n=11) in three different doses. The sham groups received daily intra peritoneal (IP) injections of the vehicle (ethanol 10%) for 30 days. Three treatment groups received different daily IP injections of the camphor (1, 2 and 5 mg/Kg) for 30 days and the control groups didn’t received anything. Serums were used for assaying LH, FSH and testosterone. The level of LH significantly increased in all doses of camphor among the treatment groups as compared to the control (p<0.05), but camphor in doses 2 and 5 mg/Kg significantly reduced the FSH level as compared to control group (p<0.05). No significant changes were seen in testosterone levels. Camphor increased level of LH, decreased level of FSH, whereas it failed to change level of testosterone. The claim of inhibitory effect of camphor on sexual activity could not be confirmed by this study. More investigations in this field are suggested.

Camphor (2-bornanon) is a ketone body from an evergreen plant of *cinamomum camphora*. It is a waxy solid with aromatic odor and is found in many medicinal plants such as thyme, lavender, chicory and date bast alcoholic extract (1-3). Synthetic derivatives are also made of pinene as well as natural kinds. Its ways of entering the body are through food, skin, eye contact and breathing. It can be poisonous when a large quantity is being ingested. It can cause irritability and neuromuscular hyperactivity, blurred vision, nausea, vomiting, colitis, dizziness, delirium, the treatment of *urinary tract infections*, contraction of heart muscles, difficulty in breathing, seizures and death (4-6). In eighteen century, camphor had been used in cumulative doses to induce convulsion attacks in psychiatric patients (7). Camphor has been used for various purposes in more countries, especially the Asian countries. In herbal medicine protocols, camphor has been administered as an antiseptic and an anti-inflammatory agent for common cold (8, 9). Camphor has been used as an additive to different substances such as ointments, lotions, as well as gels containing antiinsect bites, topical analgesic, anti-itching, anti-burns and sunburn. Also, it is used in shiny material, toilet products preservatives, cosmetics, chewing gum and cigarette (10). Recent studies showed camphor probably reduces Cytochrome P450 B1 (CYPB1) activity. This enzyme interferes in the testosterone precursors called desmolase and 17-hydroxilase. By reducing cytochrome activity, the concentration of testosterone in serum may be decreased (3). Some researchers investigated impact of camphor component in UV-filter ointment on gonadotropines and gonadal hormones, and concluded that camphor causes adolescence retardation and reduction in reproductive organs volumes in both sexes (11). Also, it was shown that the use of ointments containing camphor did not affect levels of gonadotropines [luteinizing hormone (LH), follicle-stimulating hormone (FSH)]and testosterone (12). On the other hand, it was demonstrated that camphor via effect on gonadotropin-releasing hormone (GnRH) can decrease levels of LH and FSH only at low dose. This effect may be considered as a side effect of camphor administration (13). Previous studies demonstrated the effect of camphor on abortion, breast feeding reduction and fertility suppression (3, 14, 15). Also, according to Avicenna’s belief, camphor is a suppressor of libido and acts as an effective element in reproductive system (16). Since these possible effects may be resulted in involvement of sex hormones, this study evaluated the effects of camphor on levels of LH, FSH and testosterone in the treated rats.

This experimental study was approved by the Ethics Committee of Babol university of Medical Sciences, Babol, Iran. Male Wistar rats (n=56) weighing 250- 300 gr were used. The rats were kept under standard living regimens (a 12-hour light/12-hour dark condition) and access to rat chow and water at libetum (free feeding).

Camphor was obtained from Frey+Lau Gmbh, Germany, while ethanol, diethyl ether, formalin. enzyme- linked immunosorbent assay (ELISA) kit from USCN Life Science Inc., (USA & CHINA).

Fifty six male rats were selected and divided into 5 groups including control group receiving no treatment (n=12), sham group receiving ethanol 10% (n=11) and three treatment groups (n=11) receiving 1, 2 and 5 mg/kg camphor. Ethanol 10% was used as a camphor solvent. It has been reported that ethanol in this concentration has no side effect on reproductive system (17). Base on previous studies, after 18 days of camphor administration, the rat’s blood samples were collected from orbital plexus sinus, while after 36 days of camphor administration, the blood was taken from axillaries plexus (18, 19). The samples were centrifuged and kept in the freeze condition. The serums were used for measurement of LH, FSH and testosterone using enzyme-linked immunosorbent assay (ELISA) technique.

Data were analyzed using non-parametic Kruskal- Wallis H test. The difference between the results was considered statistical significant at p<0.05.

LH levels significantly increased on days 36 (p=0.032 for 1 mg/kg, p=0.027 for 2 mg/kg, and p=0.016 for 5 mg/kg), and 18 of camphor administration (p=0.016 for 1 mg/kg). FSH levels showed a significant decrease after camphor injections on day 36 in groups receiving 2 (p=0.047) and 5 mg/kg (p=0.008) as compared to control group. No significant differences were seen in testosterone levels in all treatment groups in comparison with control and sham groups (Table 1). No significant difference was seen between sham and control groups, as well

**Table 1:** Sex hormones concentration in adult male rats after receiving daily intraperitoneal injections of camphor in three treatment groups (1, 2 and 5 mg/Kg)


Group		TSS˚ (Mean ± SD) (nmol/L)		LH (Mean ± SD) (mIU/mL)		FSH (Mean ± SD) (mIU/mL)	
		Day 18	Day 36	Day 18	Day 36	Day 18	Day 36

**Control**		0.554 ± 0.352		0.71 ± 0.301		0.084 ± 0.024	
**Sham**		0.766 ± 0.285		0.8424±0.324		0.0764 ± 0.028	
**Treatment (1)**	1 mg/Kg	0.716 ± 1.107	0.4806 ± 0.511	10.0482 ± 15.2*	1.2648 ± 0.206*	0.5466 ± 1.113	0.0702 ± 0.031
**Treatment (2)**	2 mg/Kg	0.563 ± 0.535	0.5224 ± 0.239	1.1688 ± 0.35	1.249 ± 0.228*	0.1144 ± 0.132	0.0384 ± 0.032*
**Treatment (3)**	5 mg/Kg	0.6602 ± 0.411	0.7188 ± 0.336	1.3084 ± 0.581	1.3898 ± 0.166*	0.0852 ± 0.083	0.0316 ± 0.024*


Values in table are presented as mean ± SD. TSS; Testosterone and *; P<0.05 for increased level of LH and decreased level of FSH.

These results showed that camphor significantly decreased FSH level and increased LH level in adult male rats, but did not modify the testosterone level as compared to control and sham groups. One probable reason for an increase in LH level may be due to the impact of camphor on male gonadal nerves which leads to paracrine effects on testosterone level, but it is independent of (hypothalamus-pituitary-gonad) HPG axis. According to Baumgarten et al. (20) and Rauchnwald et al. (21), these catecholamine fibers are sparsely distributed in the testis, capsule, vasculature and interstitium. Park et al. (22) and Jamshidzadeh et al. (23) have demonstrated that camphor inhibits catecholamine secretion by blocking nicotinic acetylcholine receptors. This evidence indicates the substances altering brain monoamine levels also affect reproductive system of male rat. Gong et al. (24) indicated that testicular innervation functions are considered as an important survival factor for Leydig cells *in vivo*. It appears camphor has inhibitory role in catecholamine secretion originated from testis nerves; hence Leydig cells function reduce and testosterone level decrease. On the other hand, it might be suggested that the decreased testosterone level acts on the HPG axis as a negative feedback. This can increase the level of LH. Other probable reason of increasing LH may be due to using alcohol as a vehicle in this study. This possibility is based on report of Selvage et al. (25) about inhibitory impact of alcohol on Leydig cell activity which is independent of HPG axis (24); in addition, Rivier (26) noted that alcohol induced a rapid decrease on plasma testosterone by activation of neural pathway between the brain and testis. Further studies, however, are needed to illustrate whether to use water or normal saline as vehicle. Nevertheless, paracrine control of the testis in adult men should be considered. Sharp (27) demonstrated that most cases of infertility belonged to men with raised level of serum gonadotropin despite normal or reduced sperm. Malformation of the intricated paracrein reactions within testicular tissues seem to be associated with the etiology of male infertility, particulary oligospermia.

Perhaps, in our findings, an increase in LH level showed the same effect. Another result of this research is a significant reduction of FSH level. That might be the effect of camphor on sertoli cells which inhibited FSH by inhibin secretion. The other finding of this study is the unchanged level of testosterone throughout this study despite the increased level of LH. The reason may be due to possible inhibitory effect of camphor on testis nerves and Leydig cells (22, 23). To confirm these probabil the material with a part was made of camphor ities, intracerebroventricular (ICV) injection of camphor may be helpful. There are some studies that are in conflict with our findings. Schlumpf et al. (11) investigated the impact of camphor component in UV-filter ointment on gonadotropins and gonadal hormones, and concluded camphor causes adolescence retardation and reduction in reproductive organs volumes in both sexes. Another study stated the use of ointments containing camphor did not affect on gonadotropins (LH, FSH) and testosterone (12). Also, in a quest by Carou et al. (13) effect of camphor on rats causes slightly reduce in levels of LH, FSH (only in low dose), and (GnRH) *in vitro*. It is noteworthy that they had used derivatives of camphor, or a part of used material was made of camphor, unlike our study that we used pure camphor. May be this reason describes the difference.

It is concluded that pure camphor in alcohol 10% increases LH level and decreases FSH level. To investigate other impacts of camphor on HPG axis and to confirm our results, designing a new investigation based on ICV injection of camphor is suggested.

## References

[1] Soltanipour MA, Rezai MB, Moradshahi A, Kholde Barin B, Barazandeh MM (2007). The comparison of constituents of essential oils of zhumeria majdae Rech.f.and ; wendelbo at flowering stages in various parts of Hormozgan provinc. J Med Plant.

[2] Akbarinia A, Mirza M (2008). Identification of essential oil components of Thymus daenesis Celak in field condition in Qazvin. JQUMS.

[3] Mokhtari M, Sharifi E, Moghadamnia D (2007). Effect of alcoholic extract of phoenix dactylifera spathe on histological change in testis and concentrations of LH, FSH and testosterone in male rat. IJBMS.

[4] Manoguerra AS, Erdman AR, Wax PM, Nelson LS, Caravati EM, Cobaugh DJ (2006). Camphor Poisoning: an evidence- based practice guideline for out-of-hospital management. Clin Toxicol(Phila).

[5] Zuccarini P (2009). Camphor: risks and benefits of a widely used natural product. J Appli Sci Envior Manag.

[6] Emery DP, Corban JG (1999). Camphor toxicity. J Paediatr Child Health.

[7] Pearce JM (2008). Leopold Auenbrugger: camphor-induced epilepsy-remedy for manic psychosis. Eur Neurol.

[8] Liu CH, Mishra AK, Tan RX, Tang C, Yang H, Shen YF (2006). Repellent and insecticidal activities of essential oils from Artemisia princeps and Cinnamomum camphora and their effect on seed germination of wheat and broad bean. Bioresour Technol.

[9] Chatterjie N, Alexander GJ (1986). Anticonvulsant properties of spirohydantoins derived from optical isomers of camphor. Neurochem Res.

[10] Cal K (2009). Skin disposition of d-camphor and 1-menthol alone and together. Methods Find Exp Clin pharmacol.

[11] Schlumpf M, Schmid P, Durrer S, Conscience M, Maerkel K, Henseler M (2004). Endocrine activity and developmental toxicity of cosmetic UV filters--an update. Toxicology.

[12] Janjua NR, Mogensen B, Andersson AM, Petersen JH, Henriksen M, Skakkebaek NE (2004). Systemic absorption of the sunscreens benzophenone-3, octyl-methoxycinnamate, and 3-(4-methyl-benzylidene) camphor after wholebody topical application and reproductive hormone levels in humans. J Invest Dermatol.

[13] Carou ME, Ponzo OJ, Cardozo Gutierrez RP, Szwarcfarb B, Deguiz ML, Reynoso R (2008). Low dose 4-MBC effect on neuroendocrine regulation of reproductive axis in adult male rats. Environ Toxicol Pharmacol.

[14] Edwards LF, Metoyer MS (1955). Review of methods of suppression of lactation in the puerperium and report of 108 cases treated with androgen-estrogen combination. J Natl Med Assoc.

[15] Gay WI, Heavner JE (1986). Methods of animal experimentation. Orlando: Academic Press.

[16] Avecina A (2011). Canon in Medicine.

[17] Lee HY, Naseer MI, Lee SY, Kim MO (2010). Time-dependent effect of ethanol on GnRH and GnRH receptor mRNA expression in hypothalamus and testis of adult and pubertal rats. Neurosci Lett.

[18] Hoff J (2000). Methods of blood collection in the mouse. Lab Animal.

[19] Wing TY, Christensen AK (1982). Morphometric studies on rat seminiferous tubules. Am J Anat.

[20] Baumgarten HG, Falck B, Holstein AF, Owman C, Owman T (1968). Adrenergic innervation of the human testis, epididymis, ductus deferens and prostate: a fluorescence microscopic and fluorimetric study. Z Zellforsch Mikrosk Anat.

[21] Rauchenwald M, Steers WD, Desjardins C (1995). Efferent innervation of the rat testis. Biol Reprod.

[22] Park TJ, Seo HK, Kang BJ, Kim KT (2001). Noncompetitive inhibition by camphor of nicotinic acetylcholine receptors. Biochem Pharmacol.

[23] Jamshidzadeh A, Sajedianfard J, Nekooeian AA, Tavakoli F, Omrani GH (2006). Effects of camphor on sexual behaviors in male rat. IJPS.

[24] Gong YG, Wang YQ, Gu M, Feng MM, Zhang W, Ge RS (2009). Deprival of testicular innervation induces apoptosis of Leydig cells via caspase-8-dependent signaling: a novel survival pathway revealed. Biochem Biophys Res Commun.

[25] Slevage DJ, Buchanan Hales D, Rivier CL (2004). Comparition between the influence of the systemic and central injection of alcohol on leydig cell activity. Alcohol Clin Exp Res.

[26] Rivier C (1999). Alcohol rapidly lowers plasma testosterone levels in the rat: evidence that a neural brain-gonadal pathway may be important for decreased testicular responsiveness to gonadotropin. Alcohol Clin Exp Res.

[27] Sharp RM (1986). Paracrine control of the testis. Clin Endocrinol Metab.

